# *In vivo* imaging techniques for bone tissue engineering

**DOI:** 10.1177/2041731419854586

**Published:** 2019-06-21

**Authors:** Eirini A Fragogeorgi, Maritina Rouchota, Maria Georgiou, Marisela Velez, Penelope Bouziotis, George Loudos

**Affiliations:** 1 Institute of Nuclear & Radiological Sciences and Technology, Energy & Safety (INRASTES), NCSR “Demokritos”, Athens, Greece; 2 Bioemission Technology Solutions (BIOEMTECH), Athens, Greece / Lefkippos Attica Technology Park, NCSR “Demokritos”, Athens, Greece; 3 Department of Biomedical Engineering, University of West Attica, Athens, Greece; 4 Instituto de Catálisis y Petroleoquímica (CSIC), Madrid, Spain

**Keywords:** Bone defects, single photon emission computed tomography/positron emission tomography–computed tomography imaging, healing, substitute materials

## Abstract

Bone is a dynamic tissue that constantly undergoes modeling and remodeling. Bone tissue engineering relying on the development of novel implant scaffolds for the treatment of pre-clinical bone defects has been extensively evaluated by histological techniques. The study of bone remodeling, that takes place over several weeks, is limited by the requirement of a large number of animals and time-consuming and labor-intensive procedures. X-ray-based imaging methods that can non-invasively detect the newly formed bone tissue have therefore been extensively applied in pre-clinical research and in clinical practice. The use of other imaging techniques at a pre-clinical level that act as supportive tools is convenient. This review mainly focuses on nuclear imaging methods (single photon emission computed tomography and positron emission tomography), either alone or used in combination with computed tomography. It addresses their application to small animal models with bone defects, both untreated and filled with substitute materials, to boost the knowledge on bone regenerative processes.

## Bone structure, regeneration, and grafting

The bone is a rigid organ able to support and protect various organs but is also able to facilitate mobility. These properties are mainly attributed to a remarkable hierarchical architecture of a soft collagen protein and a stiffer apatite mineral. The bone structures of different types and species are diverse at the macroscopic level and the organizations of collagen and minerals are not completely understood. However, the mineralized fibrils assembled by collagen molecules and mineralized by apatite crystals during the formation of the bone act as the bone’s universal elementary building block. The functionality of bone tissue in the body is related to stiffness, which is directly determined by the natural mineral content within the collagen/mineral composite.^1^

Bone is a highly vascularized connective tissue constituted by two-thirds of mineral components mainly in the form of crystalline hydroxyapatite (HA), calcium carbonate, and calcium phosphate. The other one-third corresponds to organic components, consisting of collagen type I (95%), proteoglycans, and other non-collagenous proteins. Bone tissue also contains growth factors (GFs), bone morphogenetic proteins (BMPs), and a significant amount of water. The proportion of organic matrix to mineral (in adult human cortical bone approximately 60% mineral, 20% organic material, 20% water) is crucial to ensure the correct balance between stiffness and flexibility of the skeleton.

Long bones are formed during embryogenesis initially as cartilage that is gradually replaced by bone. Flat bones such as the skull, by contrast, are formed directly from mesenchymal condensation. Both bone modeling (formation and shaping) and bone remodeling (replacing or renewing) occur during early childhood, whereas in adulthood bone remodeling is the predominant process that maintains skeletal integrity. The exception is the massive increases in bone formation that occur after a fracture. Most bones consist of a mixture of dense outer cortical bone and inner trabecular (spongy) bone, enabling the optimal compromise between strength and weight. In addition to providing support, attachment sites for muscles, and protection for vulnerable internal organs, bone also provides a home for bone marrow and acts as a reservoir for minerals.

**Figure 1.**
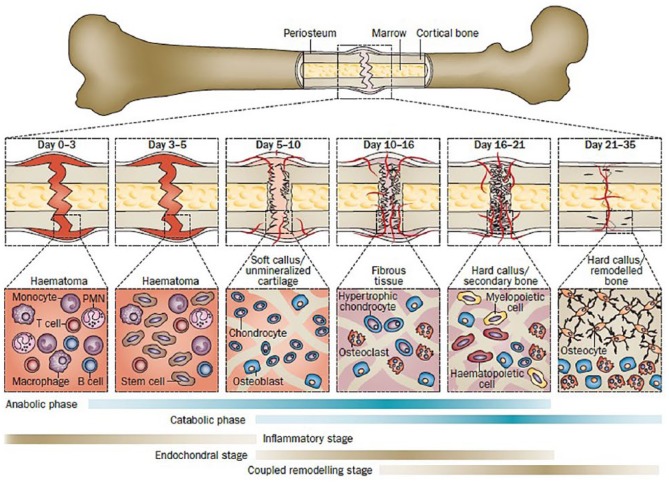
A typical healing process of a mouse femoral fracture fixed with an intramedullary rod. The major metabolic phases (anabolic and catabolic) of fracture healing are presented in the framework of three basic biological processes including inflammatory, endochondral bone formation, and coupled remodeling. (*adopted from*: Einhorn et al).^6^

Bone, unlike other tissues, can regenerate and repair itself without scars. The balanced activities of different cell types (i.e. T-cells, B-cells, stem cells, osteoblasts, osteoclasts, haematopoietic, and osteocytes) maintain bone integrity. An illustration of the healing phases of a femoral mouse fracture is provided in Figure 1. In brief, the bone-forming osteoblast produces the organic bone matrix and aids its mineralization; the bone degrading osteoclast degrades extracellular matrix (ECM) proteins and the osteocyte acts as a mechanosensor and an endocrine cell.^2–6^

Bone healing and repair usually fail in severe pathological conditions that cause extended bone defects. Insufficient blood supply, infection of the bone or the surrounding tissues, and systemic diseases can negatively influence bone healing, resulting in delayed unions or non-unions. Bone is the second most commonly transplanted tissue after blood. A bone graft is defined as an implanted material that promotes bone healing alone or in combination with other material(s), through mechanisms of osteogenesis, osteoinduction, and osteoconduction.^7^ Grafting procedures can enhance bone healing and regeneration in cases, such as cancer resection, delayed unions, non-unions, or mal-unions of fractures. An ideal bone graft material should have osteo-inducting, osteo-conducting, and osteo-integrating properties in order to be efficiently incorporated into the host bone tissue. Osteo-induction is the formation of osteoblasts stimulated and activated by BMPs, other GFs, and mesenchymal stem cells (MSCs). Osteo-conduction refers to the three-dimensional (3D) and porous material acting as a scaffold for the new bone to grow into. Osteo-integration describes the surface bonding between the host bone and the grafting material.^8–10^

Materials used in bone grafting can be divided into four main categories: autografts, allografts, xenografts, and synthetic and biologically based tissue-engineered biomaterials.^7^ The current clinical gold standard treatment is the use of autografts (the patient’s own tissue), which accounts for 58% of the bone grafts applied. However, it has major drawbacks including restricted supply and the demand of an extra operation for bone extraction. An alternative to autografts is the use of allografts (tissue from another patient), which corresponds to 34% of the current bone grafts. An alternative option is the use of human cadaver or even animal bone grafts (xenografts) which prevents complications and discomforts of the human donor site. But such types of grafts present the potential risk of viral or bacterial disease transmission or zoonoses and an immune response of the host tissue towards them.

In the last decade, tissue engineering has designed new scaffolds and tissue grafts to decrease the disadvantages of traditional grafts and improve incorporation, osteogenicity, osteoconductivity, and osteoinductivity. Thus, clinicians, with the help of researchers including engineers, molecular biologists, chemists, and material scientists, have turned their attention to designing allogenic bone substitutes with demineralized (collagen type I, osteocalcin, etc.) or mineralized bone matrix (hydroxypapatite) combined with GFs and/or MSCs to generate bone tissue. Additional strategies include developing natural-based nanocomposites^11^ and biomimetic delivery of signals^12^ to mimic artificially the structural, mechanical, spatial, and time control of the release of signals required for proper bone regeneration.

These substitute materials should be thoroughly characterized for porosity, compression and biocompatibility, biodegradation, and interaction with cells or tissues in vitro^13^ before moving to pre-clinical *in vivo* studies and clinical trials thereafter. The *in vivo* imaging techniques described in this review can be of great help for testing host graft interaction and immune response to implants, scaffolds, and viable grafts, as well as to follow signal release. This *in vivo* monitoring is essential to advance the use of tissue engineering to repair or regenerate bone tissue.^7^

## Pre-clinical imaging techniques

**Figure 2.**
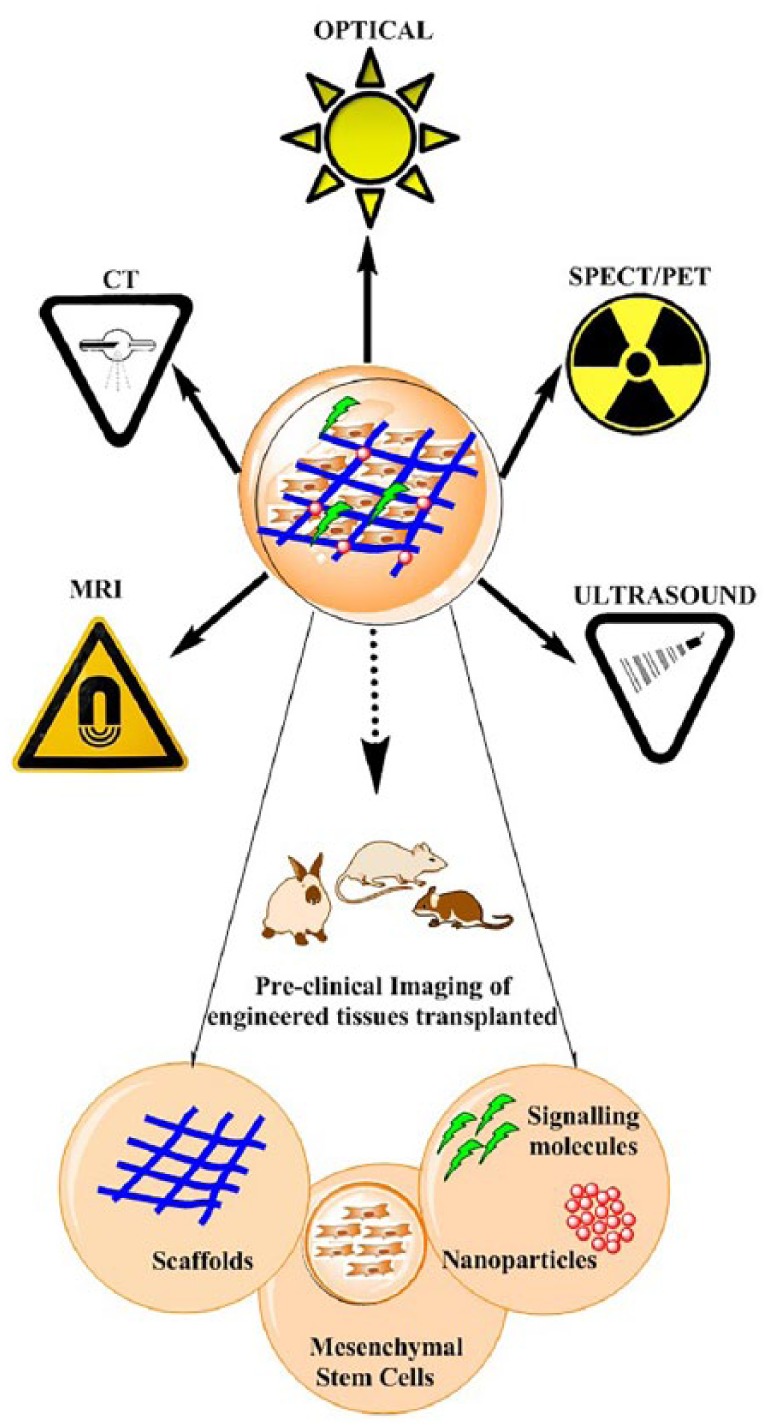
Imaging techniques with applications in bone tissue engineering.

Several real-time non-invasive imaging techniques are available to assess either bone self-healing or correct placement during implantation. They can monitor *in vivo* the natural repair and the fate of host–material interactions, as well as follow the evolution of the implanted materials over time (Figure 2). These imaging modalities can provide either anatomical (X-ray computed tomography (CT), magnetic resonance imaging (MRI), and ultrasound (US)) or metabolic (optical imaging (OI), single photon emission computed tomography (SPECT), and positron emission tomography (PET)) information on the implants.

In the next paragraphs, the main merits and limitations of nuclear (SPECT and PET) and CT imaging for monitoring bone regeneration by will be extensively discussed, and a brief overview of MRI, US, and OI imaging modalities used for bone tissue engineering (BTE) applications will be provided.

### Nuclear imaging techniques (SPECT/PET)

Nuclear (SPECT, PET) is a well-known molecular but ionizing imaging technique that relies on the detection of photons emitted from isotopes alone or combined with chemical and biologically active substances (radiotracers). No toxicity issues arise neither from the administered cold compound which, when radiolabeled, is found in trace amounts (μg–ng), nor from the radioisotope itself which can be detected even at a nano- or pico-molar level (10^−9^ M–10^−12^ M). In SPECT systems, photons from the most common gamma (γ)-emitting radioisotopes (i.e. ^99m^Tc, ^111^In, ^125^I) pass through a collimator to the detector whereas in PET devices, two annihilation photons (511 keV each) emitted in opposite directions by positron (β^+^)-emitting isotopes such a ^18^F or ^15^O are detected in time coincidence by a pair of detectors. This means that in SPECT, the spatial information between the point of emission and the point of detection is provided through the collimator (by excluding photons traveling in non-linear directions), whereas in PET the spatial information of the emission point is provided through a time window of simultaneous detection (no photons from the recorded event are excluded). Thus, PET provides higher sensitivity associated with the region of interest than SPECT and lower acquisition times. On the other hand, a unique characteristic of the SPECT imaging system is that it can acquire data using multiple energy windows at the same time, enabling the co-injection of tracers labeled with different radioisotopes for simultaneous detection of several processes.

For SPECT and PET imaging of bone regeneration, one of the drawbacks is the short half-lives of the isotopes used, which currently limits the ability to perform long-term tracking with a single radiopharmaceutical injection. Therefore, for such slow biological processes as bone repair, the radiotracer is periodically injected (usually once per week). In this case, it is more advantageous to use SPECT radiopharmaceuticals based on diphosphonates radiolabeled with ^99m^Tc, which has a longer half-life than the widely used PET bone imaging agent [^18^F]-NaF (t_1/2_ = 6 h vs 110 min) as a greater number of follow-up scans can be performed. With regard to other radioisotopes adequate for long-term PET imaging, Zirconium-89 (^89^Zr) is an attractive option, due to its favorable half-life of 3.27 days. Although ^89^Zr-labeled monoclonal antibodies have demonstrated their potential in PET imaging, for the time being there is no reference to ^89^Zr-labeled bone imaging agents in the literature. Another restriction which originates from the relatively poor spatial resolution of both nuclear imaging systems has to do with the inability to predict if the observed osteoblastic activity is related to the tissue-engineered constructs or to the host tissue itself.^14,15^

PET imaging, in contrast to SPECT, allows absolute quantification of tracer bone uptake and relative osteoblastic activity via the use of dynamic PET compartmental analysis.^15,16^ This quantitative analysis known as Hawkins method, thereafter detailed in two different studies of the “Imaging via PET/CT” section, necessitates: (a) long acquisition times over 60 min and as a consequence long-term anesthesia and (b) arterial or alternatively venous blood sampling of the animal model weekly for 6–8 consecutive weeks as bone defect healing follows a series of progressive steps. This PET [^18^F]-NaF kinetic model is a two-tissue compartment model with five parameters, four rate constants, and the fractional blood volume, also known as vessel density (VB), which corresponds to the amount of blood in the bone tissue, volume of interest (VOI). The rate constants used are for (a) tissue uptake through the hydroxyl exchange (K1), (b) reverse transport of the tracer to plasma (k2), (c) binding of the tracer to bone compartment and its fixation to fluoroapatite (k3), and (d) its release from bone compartment (k4). Within this model, the global influx of the tracer (Ki) is found using the formula: K1 × k3/(k2 + k3).

In relation to cost-effectiveness, PET instrumentation is much more expensive than SPECT as, apart from a radiochemistry laboratory for the development of the PET radiopharmaceutical, a cyclotron is needed for isotope production, which raises the overall cost even further, especially when the study lasts several weeks.

In terms of radiation exposure, the penetrating ability of PET tracers is greater than that of SPECT isotopes. In any case, the preparation of radioactive doses and the injection procedure made by persons occupationaly exposed to radiation, should be done fast and efficiently behind a thickly shielded barrier usually from lead, and a safe distance should be kept from the animal while it is being imaged.

### micro-CT

micro-CT employs X-ray attenuation measurements taken by multiple angle scans reconstructed into a 3D representation. It is an accurate, non-invasive tool providing high morphological information about bone both at a macroscopic (i.e. density, surface area) and at a microscopic level (i.e. vasculature, osteocytes) when enhanced by contrast agents or via the use of synchroton-radiation micro-CT, respectively. It is an ideal technique for longitudinal tracking of bone changes; however, there are several concerns about the long-lasting imaging process, the cumulative effects of radiation, the movement artifacts that could result in the misinterpretation of data, the efficiency of integration of bone tissue constructs within the defect area, and the clear delineation of the host tissue from the tissue-engineered construct.^17–19^

The following section will describe basic principles and features of pre-clinical nuclear imaging (SPECT and PET) modalities, combined with CT, that rely on exogenous and commercially available radiotracers for evaluation of bone repair (Table 1).^19,21,22^ Both nuclear modalities are clinically used and share advantages of whole body animal imaging, high sensitivity, while of high cost and limited in spatial resolution, as mentioned above. In this regard, for instance, spatial resolution of a pre-clinical PET system (1–2 mm) is likely worse than this of a clinical one (4.5–6 mm), as it is about a factor of three better, while the body size between a small animal (mouse) and a human differs by a factor of about 20.^22^ Therefore, most pre-clinical studies for assessing bone healing use nuclear images fused with anatomical ones obtained from CT, which provides good spatial resolution images of CT dense tissues. However, there is a raised concern about the fact that for preclinical CT imaging to obtain sufficient tissue contrast, a relatively high radiation dose must be administered to the animal, which is exposed to multiple scans for a study of longitudinal bone healing.^22^

**Table 1.** Principal characteristics of nuclear and CT pre-clinical imaging techniques in bone tissue engineering.

	CT	Nuclear	
		SPECT	PET
Spatial resolution (mm)	<0.2 mm	0.5–2 mm	1–2 mm
Probe or contrast agent sensitivity	Low (mM)	High (10^−10^–10^−11^ M)	High (10^−11^–10^−12^ M)
Penetration depth (mm)	No limit	No limit	No limit
Anatomical information	High	Poor	Poor
Equipment cost	US$200–400K	US$600–800K	US$600–800K
Radiation dose (cGy)	10–20	10–100	10–100
Imaging acquisition time	10–15 min	30–90 min	5–60 min
Probe	None for mineralized tissues like bones^20^	Gamma-ray emitting tracers ([^99m^Tc]-MDP, [^99m^Tc]-HDP or [^99m^Tc]-DPD)	Positron emitting tracer ([^18^F]-NaF)
Target	Bone tissues	Calcium ions (Ca^2+^) in HA	Hydroxyl (–OH^−^) group in HA
Advantages	High contrast among soft tissue, lung, and bone	High sensitivity to detect early bone healing reflecting the metabolic bone function	
Disadvantages	Radiation, poor contrast of bone substitute materials and between soft tissues	Radiation, poor spatial resolution	

CT: computed tomography; SPECT: single photon emission computed tomography; PET: positron emission tomography; MDP: methylene diphosphonate; HDP: hydroxy methylene diphosphonate; DPD: dicarboxy propane diphosphonate; HA: hydroxyapatite.

The tracers used to provide evidence of bone regeneration in animal defect models are based on bisphosphonates (BPs) in the form of ^99m^Tc-labeled radiopharmaceuticals ([^99m^Tc]-methylene diphosphonate (MDP), [^99m^Tc]-hydroxy methylene diphosphonate (HDP), [^99m^Tc]-dicarboxy propane diphosphonate (DPD)) for SPECT imaging and on [^18^F]-sodium fluoride (NaF) for PET imaging. In particular, BPs have high affinity for the inorganic component of bones (HA) through the binding of deprotonated oxygens of the phosphonate groups (MDP) and/or of the hydroxyl group (in case of HDP) to the Ca^2+^ ions found on bone structure. [^18^F]-sodium fluoride (NaF) accumulates to bones through the replacement of –OH^−^ groups of HA by the ^18^F^−^ ions which then migrate into the crystalline bone matrix [^18^F]-NaF has a better pharmacokinetic profile than [^99m^Tc]-MDP, including faster blood clearance and twofold higher bone uptake which reflects both bone perfusion and remodeling;^23^ however, its main disadvantage is the short half-life of ^18^F, as mentioned above. Efforts are being made to use new tracers such as peptides or other BP-based candidates for more specific tracking of bone turnover.^19,24–26^

### Other imaging techniques (MRI, US, OI)

MR imaging of bone remains challenging, due its low water content, as this imaging technique is based on the presence of a non-ionizing proton (1H) and provides high-resolution imaging of unlimited depth in soft tissues rich in water. This is the reason why structures lacking a high water percentage (such as hard bone or air) appear dark and lack a good signal in MRI. Depending on the structures that are under study, different imaging sequences can be used in MRI to highlight different tissues. The most common sequences are T1 and T2 weighted, based on the relaxation times of tissues, which differ significantly. In T1-weighted images, structures like fat, paramagnetic contrast media, melanin, or slow-flowing blood give an intense signal. T1-weighted images are produced using short echo (TE) and repetition (TR) times. Conversely, T2-weighted images are produced using longer TE and TR times. Characteristic of a T2-weighted image is the high signal intensity of water. Thus, bony structures appear dark because they give off no signal as they contain no free protons. To overcome such sensitivity limitations, several semi-quantitative MRI approaches have been applied to evaluate bone repair with the most promising being: (a) 3D spin-echo pulse sequences in combination with ^31^P nuclear magnetic resonance (NMR) spectroscopy; (b) ultra-short echo time (UTE); (c) zero echo time (ZTE); (d) sweep imaging with Fourier transformation (SWIFT); (e) use of exogenous contrast agents.^17–19^

US imaging is a high-resolution technique of low cost, portability, and lack of radiation but with poor penetration depth. This is quite evident in ultrasonic imaging systems with high frequency waves, where better resolution is attained, inversely proportionally to depth. The measured signal is based on sound waves interaction with the tissues (i.e. attenuation, absorption, reflection) and in the case of hard tissues such as bone, sound waves are well reflected. US is reflected at boundaries of different acoustic impedance, a physical property of tissues, which is dependent on the density of the tissue and the speed of the sound wave. Contrast agent (microbubble)-mediated US imaging has been applied to monitor drug therapy or gene delivery by cells^27^ and US imaging alone has been used as a diagnostic tool of comparable validity to CT for bone regeneration and for scaffold materials characterization.^19^

OI (non-ionizing) is among the oldest imaging modalities with high sensitivity but poor depth penetration that relies on photon detection and includes fluorescence imaging (FLI), fluorescence molecular tomography (FMT), and bioluminescence imaging (BLI). For FLI of bone regeneration, several commercially available exogenous fluorescent probes have been reported such as (a) high affinity, bis-phosphonate-based bone agents (i.e. pamidronate or alendronate bound to near-infrared (NIR) fluorophores of IRDye78, indocyanine green, respectively) and (b) tetracycline derivatives targeting bone linked to NIR fluorophore of IRDye 800CW. For FMT of osteoclast activity, cathepsin K targeting probe is another alternative for quantification of fluorescence in tissues.^21^ Unlike FLI and FMT, BLI needs metabolically active organisms for imaging bone repair via the use luciferase-bearing transgenic mice.^19,28^

### Bone defects in small animal models

Technology in bone tissue–engineered constructs is continuously evolving to create natural and functional bone. Despite the arising ethical dilemmas and independently of any other alternative experimentation method such as in vitro testing, animal models in BTE have a key role in the translation of such therapeutic approaches into clinics. To this respect, the in vivo assessment of bone repair first requires a surgically induced method to create the bone defect orthotopically into models. The process of bone reconstruction following an osseous injury depends on several factors such as wound defect size, anatomical site (mobility may affect the result), animal species, age and strain, bone structure, and vascularization (cortical bone and/or periosteum area).^29,30^ Healing of non-spontaneous large gaps, also known as critical sized bone defects (CSD) which are mainly caused by severe bone diseases (trauma-, tumor-, or infection-related) and other musculoskeletal deformities, is an ongoing area of research in BTE. Although no animal species is completely ideal to mimic human fracture bone disorders, the most widely used small animal models in BTE, according to peer-reviewed orthopedic articles, are rats (36%), mice (26%), and rabbits (13%).^31,32^ These models have the following ideal characteristics: throughput, reproducibility (although results can be influenced by several factors, that is, defect site and size, protocol, working site), quick healing rate, low cost and multiple types of analysis such as molecular biology, biomechanics, histological including imaging which, however, is not so practical for rabbits.^31,33^ The most widely used types of bone defects to test novel bone substitute materials or stem cell-mediated therapeutic approaches in small animals are calvarial (hole on the skull) and segmental (usually femur or tibia) defects.^13,30–32^

A summary of the most commonly used bone defects (calvarial and femoral) in small animals for testing bone substitutes via SPECT/PET and CT imaging is provided in Table 2. This table shows that rat models are the most extensively used among the species for testing through non-invasive imaging different materials, with the calvarial defect model being remarkably applied and the femoral defect one to follow. Despite the usefulness of rat calvarium in combination with SPECT/PET/CT imaging to act as a fast screening method for several grafts, it lacks load-bearing capacity and as a consequence makes it non-ideal for biomechanical tests where long bones are mainly applied. Moreover, even if for rat calvarium, the CSD is 8 mm, in seven out of nine studies smaller defects with two gaps per animal have been created. This can be explained in order to contribute to the 3R principles (Reduction, Replacement, Refinement), by minimizing the number of animals and at the same time by including both an experimental and control group in one animal, for the acquisition of more reliably compared data. However, in this case, the native potential of regeneration no longer exists.

**Table 2.** Examples of *in vivo* SPECT and/or PET, CT imaging studies of the most widely used mouse, rat, and rabbit bone defect models treated with materials/cells/tissue-engineered constructs.

Animal model	Diameter/length defect size	Implant	*In vivo* imaging analysis
Mouse			
Calvarial	4.0 mm	hASCs co-expressing BMP-2/miR-148b seeded into gelatin-coated PLGA scaffolds	μCT^34^
	3.5 mm	HA with rh BMP-2	X-ray and μCT^35^
	4.4 mm	MMP-sensitive TG-PEG hydrogels decorated with RGD peptide	SPECT/CT^36^
Femoral	0.5 mm bi-cortical trephine	ADMCs systemically injected	PET/μCT^37^
Rat			
Calvarial	8.0 mm	Chitosan gel/MSC/BMP-2	μCT^38^
	2.7 mm (on both sides of the midsagittal suture)	Collagen sponge and lactoferrin systemically injected	μCT^39^
	8.0 mm; two symmetrical gaps with 5.0 mm each	3D gelatin-based hydrogel (ArcGel) and a commercial bone graft material (BioOss)	PET/CT^40^
	Two symmetrical gaps with 4.0 mm each	rDPSCs seeded into type I collagen gel scaffolds	SPECT/PET/CT^41^
	Two symmetrical gaps with 4.0 mm each	CPC scaffolds, dense and highly porous, with PLGA particles	PET/CT^42^
	Two symmetrical gaps with 5.0 mm each	CPC/BMP-2, CPCs and an autograft material	PET/μCT^43^
		GDPB and b-TCP used alone or in combination with DPSCs	PET/μCT^44^
	Two symmetrical gaps with 5.0 mm each	(Poly (LLA-co-CL)) materials functionalised with nDPs and seeded with BMSCs	
	Two symmetrical gaps with 5.0 mm each	Murine dental pulp stem cell (mDPSC)-seeded collagen scaffolds	PET/CT^45^
Femoral	5.0 mm	PPF/TCP with DCPD	X-ray^46^
	5.0 mm	rhBMP-2 on a collagen sponge	X-ray^47^
	5.0 mm	PEGDA hydrogel combined with cells transduced with an adenovirus (Ad5) expressing BMP-2	X-ray and Μct^48^
	2.0 and 6.0 mm	PMMA	SPECT/CT^49^
	4.0 mm	CPC enriched with strontium (SrCPC)	dPET/CT^50^
	4.0 mm	CPC, collagen/silica and iron composites	dPET/CT^50^
	3.0 mm	Silastic spacer	PET^51^
Rabbit			
Calvarial	8.0 mm (two parietal defects)	ASCs engineered to express BMP-2 or TGF-b3 in PLGA and gelatin constructs	PET/CT^52^
Femoral	6.0 mm	Magnesium	X-ray^53^
	15.0 mm	BMP-2-derived oligopeptide P24 in combination with PLGA-[ASP-PEG] scaffold	X-ray^54^

SPECT: single photon emission computed tomography; PET: positron emission tomography; CT: computed tomography; ASCs: adipose-derived stem cells; PLGA: poly(lactic-co-glycolic acid; HA: hydroxyapatite; rh: recombinant human; BMP: bone morphogenetic proteins; MMP: metalloprotease; TG-PEG: system from poly(ethylene glycol)-based macromer via the transglutaminase factor XIII; RGD: Arginylglycylaspartic acid, Arg-Gly-Asp; ADMCs: adipose-derived multipotent cells; MSC: mesenchymal stem cells; rDPSCs: rat dental pulp stem cells; CPC: calcium phosphate cement; GDPB: granular deproteinized bovine bone; b-TCP: beta-tricalcium phosphate; DPSCs: dental pulp stem cells; poly (LLA-co-CL, poly (l-lactide-co-ε-caprolactone); nDPs: nanodiamond particles; BMSCs: bone marrow stromal cells; mDPSC: murine dental pulp stem cell; PPF/TCP: polypropylene fumarate/tricalcium phosphate; DCPD: dicalcium phosphate dehydrate; PEGDA: poly (ethylene glycol) diacrylate; PMMA: poly-methyl-metacrylate; TGF-b3: transforming growth factor b3; PLGA-[ASP-PEG]: poly (lactic acid/glycolic acid/asparagic acid-co-polyethylene glycol).

In the following section, we will mainly focus on the in vivo SPECT or PET imaging studies relying on the widely used bone imaging tracers in combination with CT, which are applied in pre-clinical models. However, there are a few studies where bone regeneration is tracked using radio-iodinated BMP-2 embedded in scaffolds.^55–57^ Notwistanding its usefuleness, such a method cannot provide direct information about bone formation, but gives only a hint of the presence of BMP-2.

### Imaging via SPECT/CT

Skaliczki et al.^49^ studied the bone healing of a Wistar rat femoral defect model using a NanoSPECT/micro-CT imaging system (Mediso Ltd-Bioscan Inc, Hungary, USA) and the radioactive probe ^99m^Tc-MDP. The main purpose of this study was to establish a reliable experimental bone defect model by creating either a gap of a critical size (6 mm) or of a non-critical one (2 mm) that would allow the screening of novel bone implants. For blocking healing rate, an interposition spacer was inserted in the group of animals with the non-critical size gap. The uptake of ^99m^Tc-MDP, a specific label for osteoblastic activity, was higher at the operated mid-diaphysis part compared to the intact one mainly at 7 days after surgery. The healing rate at 4 weeks was only 12.5% in CSD animals, ~83% in animals without spacer and of the same ratio in animals where an interposition technique was applied, and the follow-up period was extended for 4 weeks after spacer removal.

**Figure 3.**
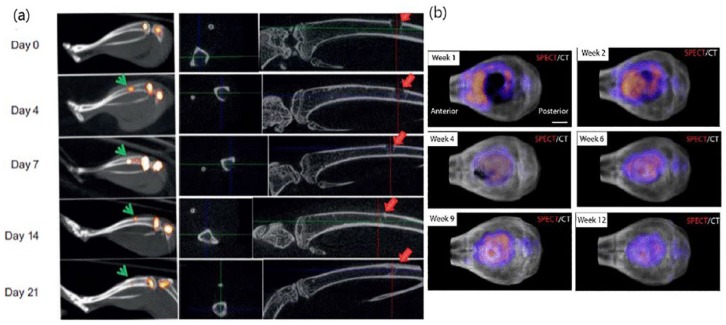
(a) Representative SPECT (left panel) and CT (right panel) of injured tibiae in mice at different time points (adopted from Zhong et al.^58^) and (b) merged SPECT/CT images of calvarial defect mice treated with BMP-2 loaded hydrogels (adopted from Lienemann et al.^36^).

Zhong et al.^58^ demonstrated that ^99m^Tc-MDP uptake corresponding to osteoblastic activity reached a peak on day 7 and started decreasing on day 14 post-injury (Figure 3(a), left panel). The experiments were done through imaging of a skeletal injury transgenic mouse model (Ocn-Cre; mT/mG) with a hole of 1 mm in the proximal tibia on a NanoSPECT/CT scanner (Bioscan, Washington, DC, USA). Bone healing and mineralization according to ex vivo CT data started from day 7 and reached a peak on day 14 post-injury, which coincides with the information obtained from the nuclear imaging experiments. In the same study, ^99m^Tc-MDP uptake at calvarial cells differentiated into osteoblasts (days 0, 4, 7, 14, and 21) was also investigated by the SPECT scanner and it was shown that it was increased along with osteoblast marker Ocn expression, as confirmed by real-time polymerase chain reaction (PCR). In this case, ex vivo, in vitro and even molecular biology techniques gave the same information as in vivo imaging.

Lienemann et al.^36^ evaluated bone regeneration using a NanoSPECT/CT imaging system (Bioscan, Washington, DC, USA) in a calvarial bone defect (4.4 mm) C57Bl/6 mouse model after implantation of PEGylated, RGD-functionalized and BMP-2 (loaded or not) hydrogels (Figure 3(b), right panel). Both bone formation and volume, as evaluated by micro-CT, started increasing at week 2 post-surgery at the defect area, attaining a peak at 4 weeks without significant change up to 12 weeks. In contrast, SPECT imaging in mice treated with BMP-2 loaded hydrogels gave an earlier signal, showing high ^99m^Tc-MDP uptake in the bone defect during the first week post-treatment that increased at week 2 around the defect area and in its center from week 4 to week 12. These results show that the higher sensitivity of SPECT provides a high prognostic value for bone healing.

Ventura et al.^41^ used ^99m^Tc-HDP to weekly monitor (for a period of 10 weeks) only untreated bone defects (two symmetrical gaps of 4 mm width) in a Wistar rat calvarial model by SPECT/CT. Radioactivity reached a peak at 4 weeks post-surgery around the limits of the defect area. From CT scans, the healing of the defects started at week 3 and was accomplished at week 10 post-surgery. Thus, SPECT revealed the earliest signs of new bone tissue deposition and CT provided a clear visualization of the healing process over time.

Zhou et al.^59^ used SPECT and X-ray imaging to reveal bone metabolism and implant vascularization of ulnar bone defects (1.5 cm) in rabbits following repair with β-tricalcium phosphate (β-TCP) ceramic constructs combined with MSCs and/or MSC-derived endothelial cells (ECs). A SPECT study performed at 4, 8, and 12 weeks post-surgery showed high ^99m^Tc-MDP uptake indicating better osteogenesis results, with the β-TCP constructs enriched by co-cultured MSCs and MSC-derived ECs when compared to the same β-TCP implants alone or combined with MSCs. However, even though the signal in the defect area increased with time, the rate of ratio uptake of treated to non-treated areas decreased from 8 to 12 weeks after surgery. Through the X-ray imaging study carried out at 4, 8, 12, and 16 weeks post-surgery, there were indications for new bone formation and marrow cavity recreation at weeks 4 and 8 post-surgery, in case of the β-TCP constructs embedded with mesenchymal cells (MSCs or MSC-derived ECs). Signs of partial and full repair were visible at week 16 for animal groups of b-TCP (+) MSCs and b-TCP (+) MSCs (+) ECs, respectively. Thus, both SPECT and X-ray imaging demonstrated that grafts of MSCs co-cultured with ECs enhanced osteoblasts proliferation and local vascularization of bone tissue rapidly within the first 8 weeks and then steadily up to week 12.

The pore structure of the scaffold plays a critical role for proper cell growth and migration, nutrient circulation, and vascularization. Therefore, Bai et al.^60^ tested in vivo the vascularization of porous b-TCP-based bioceramic materials (with several pore sizes (300–700 μm) and interconnections (70–200 μm) between pores) implanted in fascia lumbodorsalis of rabbits, via SPECT imaging. To this extent, after rabbits were injected with ^99m^Tc-MDP and according to the highest radioactive signal obtained at the area of interest mainly at 4 and 8 weeks post-surgery, better vascularization was succeeded with implant pore size up to 400 μm and an interconnection pathway of 120 μm.

It is difficult to somehow draw a general conclusion form the above-mentioned SPECT/CT imaging studies as there are many differences among the animal model, the defect site, the type of materials used, and the imaging systems applied by each research group. Nonetheless what can be safely deduced is that SPECT imaging, when compared to CT, provided an earlier indication of any bone healing effect which was enhanced in the presence of biomaterials and was further boosted when material scaffolds were combined with GFs or cells, without neglecting the fact that the porous structure of such materials plays a significant role of their revascularization.

### Imaging via PET/CT

An ideal scaffold in BTE should enhance cell viability, adherence, proliferation, osteogenic differentiation, vascular ingrowth, host integration, and load bearing.^61^

Ventura et al.^41^ used [^18^F]-NaF to monitor bone regeneration in a rat calvarial defect model consisting of two symmetrical gaps of 4 mm width. Two kinds of calcium phosphate cement (CPC) scaffolds were tested, either dense or porous (with poly (DL-lactic-co-glycolic acid)–PLGA particles). [^18^F]-NaF activity uptake increased from week 2 up to week 6 post-implantation and, was higher in the porous scaffolds than in the dense ones. The CT study showed that the detection of the healing process via the use of scaffolds was not so apparent. When PET metabolic data obtained for untreated defects were compared to SPECT data, the former were found to be of much higher sensitivity; namely [^18^F]-NaF showed faster (even from day 1 after surgery) and greater (at all time points) uptake than the technetium bone imaging tracer with optimum uptake at week 4 after surgery. On the whole, higher activity was detected in the defect area in the presence of the porous scaffolds than of the dense ones, limiting their superiority in relation to solid materials, while CT made the healing process over time anatomically visible.

**Figure 4.**
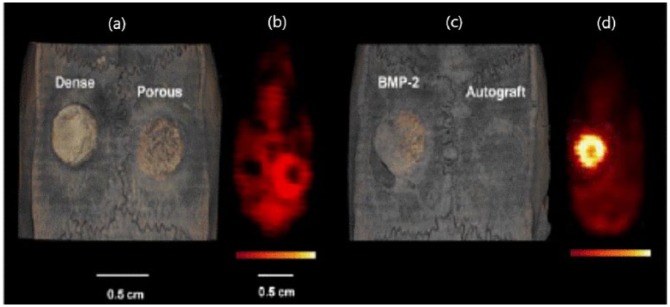
μCT (a, c) and PET (b, d) images of the four experimental groups (two groups per animal) at 4 week post-implantation. (*adopted from*: Ventura et al).^42^

The same group^42^ assessed bone morphogenic protein-2 (BMP-2) release from CPC scaffolds by PET/CT after injection of [^18^F]-NaF CPCs (dense and highly porous), autograft implants used as controls (Figure 4). PET fused with CT imaging started at day 2 and was then performed every 2 weeks up to week 8 post-surgery in Wistar rats with a cranial defect (two symmetrical holes of 4 mm width). Animals with BMP-2 loaded CPCs displayed enhanced radioactivity uptake values maintained up to week 4 post-surgery, which was much higher and more homogeneously distributed throughout the implant area compared with the three control animal groups. A significant dip in signal at week 6 after surgery was noted in all studied groups. By CT imaging, new bone formation was only detectable at week 4 post-surgery in animals implanted with the BMP-2 loaded and empty porous scaffolds, although quantification was not feasible as they were of equal radiopacity with natural bone. Thus, CT was mainly used as a standard for anatomic information while PET depicted the higher new bone formation in the BMP-2 releasing group, compared with the controls.

When combined with a scaffold, MSCs of different origin (i.e. adipose tissue- (ADPCs), bone marrow- (BMCs), and dental pulp- (DPSCs) derived stem cells)^62–64^ are a promising alternative to the use of bone implants alone for not only sustaining cell growth, but also for responding to biological stimuli in order to release impregnated GFs and interact with the tissue environment to induce bone repair.

To this respect, Annibali et al.^43^ used μCT and PET imaging (through [^18^F]-NaF) to study bone regeneration induced by commercially available biomaterials (granular deproteinized bovine bone (GDPB) and b-TCP) implanted alone or in combination with dental pulp stem cells (DPSC) in a 5 mm (two symmetrical gaps) rat calvarial defect model, at 4 and 12 weeks after implantation. According to this study, the presence of stem cells did not increase the signal (low standardized uptake values (SUVs)) around the defect area for either type of scaffold. However, GDPB seemed to better enhance bone regeneration, which is either attributed to its correct placement to the gap or to the scaffold itself.

Lee et al.^37^ used [^18^F]-NaF for micro-PET imaging of adipose tissue-derived multipotent cell-mediated (ADMC) bone healing effect in femoral bone defect mice (0.5 mm bi-cortical trephine defect) and in mice without trephination of the femur (sham-operated animals). [^18^F]-FHBG (9-fluoro-hydroxy-methyl-butyl-guanine) was also used to verify if the systemically administered cells were concentrated at the injured area. A statistically significant increase of [^18^F]-NaF uptake in injured femur compared to the intact contralateral femur was observed on days 4 and 10 post-surgery, but the intensity of the activity was not significantly different between the two bone injury groups of intravenously cell-injected and non-injected mice. It was demonstrated that [^18^F]-FHBG was not specifically bound to the stem cell-associated femoral wound. By micro-CT imaging, bone healing of the trephine defect started earlier (from day 7 post-surgery) in cell-injected animals than in non-injected ones, which displayed a comparable healing level on day 14 post-operation.

Another BTE approach based on stem cell therapy combined with scaffolds was studied by Collignon et al.^45^ who evaluated via PET/CT imaging the potential of detecting any early signs of angiogenesis and bone healing induced by murine dental pulp stem cell (mDPSC)-seeded collagen scaffolds. Two symmetrical gaps with a diameter of 5 mm each were created at the skull of a rat model and were left either empty or were filled with normoxia mDPSC-seeded (group a), 5 (%) hypoxia-primed mDPSC-seeded (group b) and acellular scaffolds (group c). For assessing early angiogenesis and bone healing via nuclear imaging, [^64^Cu]-NODAGA-RGD and [^18^F]-NaF were used as tracers, on day 10 and on days 45 and 90 post-surgery, respectively. For both tracers, the highest uptake was observed in the defect area filled with the hypoxic primed scaffolds, which seemed to enhance the formation of new vessels and thereafter the healing process. μCT analysis done on days 30 and 90 post-surgery also showed that mDPSC-seeded scaffolds exerted significantly high osteogenic effect, with the primed ones giving even better results.

Yassin et al.^44^ evaluated the potential of poly (l-lactide-co-ε-caprolactone) scaffold (poly (LLA-co-CL)) materials functionalised with nanodiamond particles (nDPs) and seeded with bone marrow stromal cells (BMSCs). Thus, osteogenic activity was assessed in a 5 mm (two symmetrical holes) rat calvarial model at 4 and 12 weeks post-implantation of both poly (LLA-co-CL)/BMSCs and poly(LLA-co-CL)/nDPs/BMSCs after [^18^F]-NaF administration, on a small animal PET/CT imaging system provided by Mediso. It was thus demonstrated that tracer uptake at the defect area was low at 4 weeks post-surgery for both types of implanted materials, while it significantly increased in the presence of poly (LLA-co-CL)/nDPs/BMSCs scaffolds at 8 weeks post-surgery. This was also confirmed by the findings via μCT, where higher bone volume was observed after treatment with the nDP-functionalized scaffolds.

As bone healing involves a dynamic interplay of biological processes, dynamic PET-CT imaging is considered a suitable tool for real-time tracking of the extent and rate of bone formation. To this respect, Cheng at al.^50^ applied [^18^F]-NaF for dynamic PET-CT (dPET-CT) evaluation of the healing of femoral (metaphyseal area) bone defects (4 mm) in osteoporotic (induced by ovariectomy and a diet free of Ca, Vitamin D, etc.) rats with CPC only or combined with strontium (SrCPC). PET/CT imaging was performed once, at week 6 after surgery, where the highest uptake was observed in the SrCPC-treated defect. For more quantitative information, two kinetic parameters were measured, k3 (representing the formation of fluoroapatite via the exchange of –OH^−^ groups of HA crystal with F^−^) and vessel density (VB (corresponding to the amount of blood in the VOI) and it was demonstrated that k3 values were greater in both biomaterial-implanted animals related to non-treated animals and were even higher in the SrCPC rats, while VB values were lower in the treated animals than in ones with no biomaterial.

Dynamic PET-CT imaging was again used by Cheng at al.^50^ to monitor the healing effect of three different types of biomaterials based on CPC, collagen/silica and iron composites, in 4 mm femoral defect rat osteoporotic models 6 weeks after surgery. For quantitative evaluation, SUVs were determined and a two-tissue compartmental model was applied to the data for the calculation of the kinetic parameters (k1–k4, VB, ki). For [^18^F]-NaF, k1 and k2 represent the F-exchange with OH^−^ groups of HA crystal of bone and the reverse process, respectively, while k3 and k4 represent the formation of fluoroapatite and the opposite. The most sensitive PET parameter k3, which is indicative of ossification tested at CPC and SrCPC based materials showed that both enhanced bone formation, although SrCPC exhibited a higher rate of osteogenesis. k3 values also measured at collagen and silica composites were lower for silicate/collagen samples (B30) than those in the form of scaffolds (Sc-B30), indicating that new bone formation is stimulated in the presence of porous biomaterials. No significant difference was observed in the defect area filled with the silica/collagen xerogel scaffolds combined with strontium (Sc-B30Sr20) in relation to B30 samples. Ki value, describing the net plasma clearance of [^18^F]-NaF to the bone mineral, measured at iron foam implants, was significantly higher in the presence of iron foam coated with zolendronic acid (Fe-BP) than with Fe only.

Hsu et al.^51^ compared the use of [^18^F]-NaF and [^18^F]-FDG for assessing fracture healing in rat femoral models via PET imaging at weeks 1, 2, 3, and 4 after surgery. Two animal groups were evaluated, the first one with a manual three-point bending technique and the second one with osteotomy using a 3 mm drill and then covered by a silastic spacer. [^18^F]-NaF scans showed that SUVs were significantly higher in the first group when compared to the second group at all time points tested. Bone healing in the first group started even from week 1 and SUVs attained a peak between weeks 3 and 4. [^18^F]-FDG uptake at fracture sites of successful and delayed bone healing did not provide any extra information and its diagnostic role regarding fracture non-unions was questionable.

Even if [^18^F]-FDG has limited usefulness in following healing process of bone defect animal models, it could be helpful in the diagnosis of post-operative infection and inflammation. In this respect, Lohmann et al.^40^ to test the osteogenic potential of a 3D gelatin-based hydrogel (ArcGel) scaffold compared it with a commercial bone graft material (BioOss) and an autograft and used both [^18^F]-FDG for assessing bone metabolism correlated to inflammation response and [^18^F]-NaF for bone healing. In this study, a critical size (8 mm) calvarial defect model in rat was used and μPET/CT imaging was performed at days 1, 3 and at weeks 3, 6, and 12 after surgery. The highest glucose metabolism for both ArcGel and BioOss was observed at day 1 post-surgery, while the highest osteoblastic activity for all scaffolds was at day 3, remaining steady up to 3 weeks, although for BioOss it was still high even at 12 weeks post-surgery. According to μCT imaging, the gap filled with ArcGel was almost covered at 3 weeks post-surgery, but in the case of BioOss and autograft there was not a discrete difference between the implant and the newly formed bone. However, closing of the defect area for all tested scaffolds was observed at 12 weeks post-surgery, which is also confirmed by ex vivo CT measurements.

To sum up, PET imaging of the osteoblastic activity at the defect area gave images of higher contrast than SPECT, which can be attributed either to the better spatial resolution of PET scanners or to the fact that [^18^F]-NaF kinetics are not affected by protein binding as in the case of [^99m^Tc]-MDP. The above data further indicated that bone healing efficiency was improved by the incorporation of stem cells and GFs into scaffolds, and that quantitative PET imaging of bone turnover is indeed a suitable approach for measuring the mineral apposition rate. Finally, even if [^18^F]-Fluoro-deoxyglucose (FDG) is not so specific for visualizing bone turnover, it could be applied as a marker of inflammation/infection associated with bone defect creation in animal models.

## Concluding remarks

Nuclear imaging applied to the study of bone regeneration is gradually evolving and due to the use of combined SPECT or PET/CT imaging systems, the specificity of applied tracers has significantly improved. These hybrid techniques complement the gold standard imaging modality (CT), contributing in particular to efficiently monitoring early healing effects of a synthetic bone substitute applied to large bone defects, where information acquired by CT is not so accurate. This is due to the low sensitivity of CT to capture low-density mineralized deposition or to its lack of capacity to distinguish scaffolds from original bone, both of them with similar radiopacity. Another reason is that molecular imaging provides additional functional information of bone metabolism, predicting the success of a therapeutic scheme from a very early stage. However, CT scans are useful for anatomical reference and for tracking the original defects.

Therefore, pre-clinical nuclear imaging combined with CT is evolving to become a key technology to track bone tissue–engineered constructs, providing real-time quantitative information on biological processes non-invasively, cost-effectively and with high sensitivity and resolution, thus playing a fundamental role in translation of tissue engineering developments to the clinic. It is worth mentioning that the ability of dynamic PET imaging to quantitatively monitor the response to BTE treatment after surgical intervention renders it superior with regard to SPECT, even if such an approach has limited clinical applications.
